# Closing the Door with CRISPR: Genome Editing of CCR5 and CXCR4 as a Potential Curative Solution for HIV

**DOI:** 10.3390/biotech11030025

**Published:** 2022-07-14

**Authors:** Julian J. Freen-van Heeren

**Affiliations:** Independent Researcher, Amsterdam, The Netherlands; j.freenvanheeren@gmail.com

**Keywords:** CRISPR, HIV, gene editing, CCR5, CXCR4

## Abstract

Human immunodeficiency virus (HIV) infection can be controlled by anti-retroviral therapy. Suppressing viral replication relies on life-long medication, but anti-retroviral therapy is not without risks to the patient. Therefore, it is important that permanent cures for HIV infection are developed. Three patients have been described to be completely cured from HIV infection in recent years. In all cases, patients received a hematopoietic stem cell (HSC) transplantation due to a hematological malignancy. The HSCs were sourced from autologous donors that expressed a homozygous mutation in the *CCR5* gene. This mutation results in a non-functional receptor, and confers resistance to CCR5-tropic HIV strains that rely on CCR5 to enter host cells. The Clustered Regularly Interspaced Short Palindromic Repeats (CRISPR)/CRISPR-associated (Cas) system is one of the methods of choice for gene editing, and the CRISPR/Cas system has been employed to target loci of interest in the context of HIV. Here, the current literature regarding CRISPR-mediated genome editing to render cells resistant to HIV (re)-infection by knocking out the co-receptors CCR5 and CXCR4 is summarized, and an outlook is provided regarding future (research) directions.

## 1. Introduction

While the diagnosis, treatment and monitoring of individuals infected with human immunodeficiency virus (HIV) has progressed massively since the identification of HIV as the causative agent of acquired immunodeficiency syndrome, or AIDS, in the early 1980s [1,2,3,4], unfortunately, a definitive cure has as of yet not been developed. HIV infection can typically be controlled by anti-retroviral therapy (ART) if patients are meticulous in adhering to dosing regimens and regular follow-ups. However, ART is not without risks, as HIV-infected individuals treated with ART are at greater risk of, e.g., fractures, central nervous system disorders and diseases of the cardiovascular, liver and renal systems [5]. Therefore, considering these serious adverse events, it is imperative that (permanent) cures for HIV-infected patients are developed.

HIV-mediated destruction of the immune system is mostly driven by cellular tropism [6,7,8]. For cellular entry, HIV requires CD4 and a co-receptor, with CCR5 and CXCR4 being the most common [9]. Therefore, the main cellular targets of HIV infection are CD4^+^ T cells and monocytes [6]. After successful infection, infected cells are instructed to actively produce new virions, or function as a safe haven, from which HIV can re-emerge after a period of latency [10]. As such, treatment should focus on either eradicating cells harboring the HIV genome, or rendering cells refractive to (re)-infection, or, ideally, both.

HIV relies on host factors for infection and propagation, but is especially reliant on the expression of co-receptors to recognize and invade target cells. Therefore, a part of the solution to the HIV cure could lie in rendering target cells invisible to the virus. In recent years, three patients have been described to be completely cured from HIV infection [11,12,13]. Interestingly, in all cases, patients received a hematopoietic stem cell (HSC) transplantation due to a hematological malignancy. The HSCs were sourced from autologous donors that expressed a homozygous mutation in the *CCR5* gene. This mutation, also known as CCR5Δ32/Δ32, results in a non-functional receptor, and confers resistance to CCR5-tropic HIV strains [14]. While one patient died as a result of the hematological malignancy [12], the other two have undetectable viral loads after being reported to be off of ART for more than 3.5 years [15] and 30 months [16], respectively. However, while this approach sounds promising, only ~1% of the Caucasian population is CCR5Δ32/Δ32, with even lower frequencies reported in people from Asian and African descent [17]. As finding a suitable Human Leukocyte Antigen (HLA)-matched donor is difficult, the odds of finding a HLA-matched CCR5Δ32/Δ32 donor within the current HSC donor pool are very small [18]. Therefore, additional sources of CCR5Δ32/Δ32 HSCs are required, or alternatively, not expressing CCR5, as low CCR5 expression has been shown to be protective in elite HIV controllers compared to a control group of HIV-infected individuals [19].

One way of increasing the donor pool would be through genetic modification. Several gene editing tools have been employed in the battle against HIV, including Zinc Finger Nucleases (ZFNs), Transcription Activator-Like Effector Nucleases (TALENs) and the Clustered Regularly Interspaced Short Palindromic Repeats (CRISPR)/CRISPR-associated (Cas) system [20,21,22,23,24,25,26,27]. While each tool has their advantages and disadvantages, the main benefit of using the CRISPR/Cas system is the limited off-target effects [28,29] and broad genome targeting capacity [30]. CRISPR has successfully been employed as a gene editing tool in clinical trials [31], and a trial is currently planned/in progress investigating the use of CRISPR-mediated CCR5 knockout cells in the context of HIV (ClinicalTrials.gov identifier NCT03164135).

In this review, first, a brief overview of the HIV infection cycle will be provided to discuss targets suitable for gene editing. Next, the different genome editing tools will be discussed. Then, the current literature regarding CRISPR-mediated genome editing to render cells resistant to (re)-infection is reviewed, and an outlook is provided regarding future (research) directions.

## 2. The HIV Infection Cycle—Targets for Therapy?

**Figure 1:**
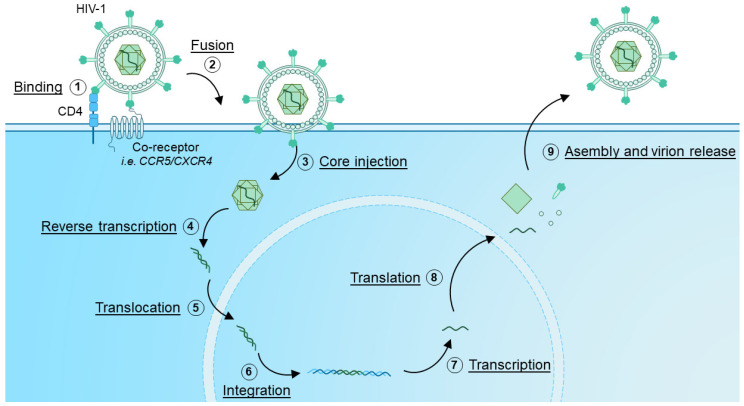
HIV infection and replication cycle. (1) HIV binds to target cells expressing CD4 via interaction with Envelope glycoproteins present on the virion. Co-receptors, such as CCR5 and CXCR4 are also engaged, which co-receptor is engaged is dependent on viral tropism. (2) By inserting the HIV Envelope glycoprotein into the membrane, the fusion of the virion and host cell membrane is enforced. Next, (3) the HIV viral core is injected, after which (4) the HIV viral genome is reverse transcribed and (5) translocated into the nucleus. (6) The HIV genome, now in DNA form, integrates into host DNA, where it can serve as a template for (7) transcription and (8) translation. The newly transcribed RNA and viral proteins are then (9) assembled into new virions which are released from the infected cell.

The exact mechanisms of HIV infection, propagation and viral particle formation have been extensively reviewed elsewhere [32,33,34], but, briefly, during a primary infection, HIV utilizes envelope glycoproteins to bind Cluster of Differentiation (CD)4 on target cells [32] (Figure 1). Additionally, co-receptors are engaged, with the chemokine receptors C-C Motif Chemokine Receptor 5 (CCR5) and C-X-C Motif Chemokine Receptor 4 (CXCR4) being the most common [9,32,35]. Therefore, the main cellular targets of HIV infection are CD4^+^ T cells and monocytes [6]. Next, HIV employs part of the HIV envelope protein, glycoprotein gp41, by inserting it into the membrane of the target cell to facilitate fusion of the viral envelope and the target cell membrane [34]. This allows for delivery of the viral core [36], containing the genetic information of HIV as a Ribonucleic Acid (RNA) template. The HIV genome is subsequently reverse-transcribed by the reverse-transcriptase enzyme into Deoxyribonucleic Acid (DNA) to allow insertion into the host genome [37]. This inserted DNA then forms the blueprint for the transcription and translation of HIV proteins, and assembly of new viral particles [33]. The new HIV virions are subsequently released by infected cells, releasing them into the extracellular space, where new target cells can be infected and turned into virion-producing factories.

While several host-proteins function as well-characterized restriction factors, such as APOBEC, TRIM5 and SAMHD1 [38], studies have also shown host-factors on which HIV is dependent. For instance, Rebensburg et al. showed that HIV-1 depends on Sec24C for replication through interactions with the viral core, and is important for reverse transcription, nuclear import and infectivity [39]. Other studies have similarly investigated other intracellular targets that are important for HIV infection and replication after cellular entry [40,41].

However, preventing HIV from entering target cells would allow to prevent cellular infection in the first place. As indicated in Figure 1, HIV requires both CD4 and a co-receptor to facilitate entry. CD4 is a crucial co-receptor for TCR-mediated T cell activation. While it remains unclear whether it is required for stabilization of peptide-major histocompatibility complex (TCR interactions), it does function as an anchor for important kinases such as Lck that are required for TCR-mediated T cell activation [42]. Therefore, CD4 is not a desirable nor druggable target. However, HIV also requires co-receptors in order to facilitate central entry. As discussed, CCR5 and CXCR4 are the best described co-receptors [9,32,35]. Combined with the fact that the CCR5Δ32/Δ32 mutation confers resistance to infection [14,43], these molecules could be attractive targets as a potential cure, and as such have been investigated as targets to render cells refractive to HIV entry [44,45]. Therefore, the remainder of this review will focus mostly on these two targets.

## 3. Gene Editing Basics—Mode of Action and Different Tools

As discussed, several gene editing tools are available for genome editing. In this section, the three most prominent gene editing tools (ZFNs, TALENs and CRISPR) and their advantages and disadvantages will be described.

ZFNs are fusion proteins, consisting of site-specific DNA-binding domains isolated or adapted from zinc-finger-containing transcription factors fused to the endonuclease domain of the bacterial FokI restriction enzyme [30]. For gene editing to occur, ZFNs targeting both the positive and negative strand are required [30]. As the targeting domain is incorporated into the ZFN, novel targeting domains need to be developed and tested for targeting new genomic regions. Through academic and commercial efforts, tools and libraries exist that can be used to target genomic sequences every 50–200 base pairs [30].

Similarly, TALENs also rely on the DNA-binding domain encoded into the TALEN, which can be used to target a specific locus of interest, and like ZFNs, TALENs rely on FokI to induce DNA double strand breaks [30]. DNA recognition is based on a variable residue that specifically recognizes a DNA base [30]. As such, TALENs can be produced that specifically recognize a stretch of DNA by incorporating the correct variable residue sequence [30].

**Figure 2:**
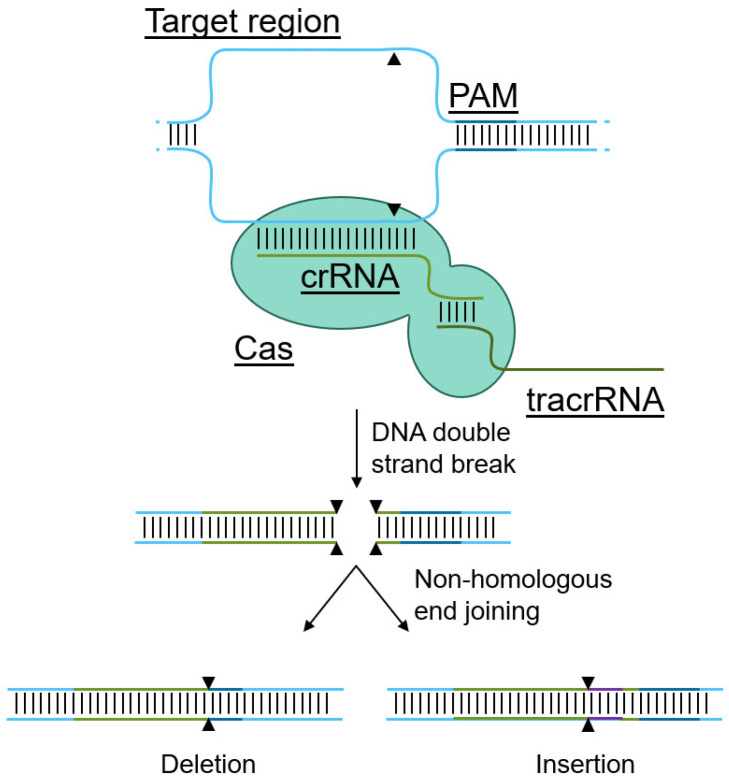
Mechanism of CRISPR-mediated genome editing and delivery methods. Cas proteins utilize a targeting crRNA to recognize the target site in the genome, but requires a trans-activating tracrRNA to induce a double strand break. The resulting double strand break is repaired via non-homologous end joining, resulting in deletions and insertions, effectively rendering genes non-functional.

In contrast, the genomic targeting of the CRISPR/Cas system is not encoded directly into the Cas protein. Instead, the genomic locus of interest is identified by an accessory RNA molecule (Figure 2) [46]. Indeed, CRISPR/Cas-mediated genome editing relies on three components: (1) the Cas protein, a DNA nuclease that can be targeted to a region of interest with (2) a targeting CRISPR RNA (crRNA), but requires (3) a trans-activating CRISPR RNA (tracrRNA) that facilitates activation of the Cas catalytic activity, inducing a DNA double strand break upon recognition of the binding sight [46]. Subsequent non-homologous end joining results in the introduction of insertions and deletions, effectively altering the DNA sequence, often leading to gene knockouts. To prevent random catalytic activity and to enhance on-target cleavage, Cas-proteins require the presence of a protospacer-adjacent motif (PAM), directly adjacent to the targeting crRNA site [28]. This PAM is specific to each Cas protein, and can be anywhere between 3–8 base pairs [46,47]. Without this PAM, the activation of the Cas-protein by the trans-activating CRISPR RNA is much less efficient, if effective at all [28], thereby limiting off-target effects. Of note, several optimization strategies can be considered to limit off-target effects, such as alteration of the crRNA and optimizing Cas9 concentrations [29]. While a PAM needs to be directly adjacent to the intended cleavage site to allow CRISPR-mediated editing [28], due to the variety in naturally occurring Cas-proteins and Cas-proteins with mutations in the PAM-recognition domain, a variety in PAM sequences is available to facilitate gene targeting [28,48,49,50]. As a result, virtually any gene or DNA region of interest can be targeted with the CRISPR system, in contrast to other gene editing tools such as ZFNs [30]. Due to these advantages, the remainder of this manuscript will focus on CRISPR as the gene editing tool of choice to target CCR5 and CXCR4.

Several methods have been designed to introduce the CRISPR/Cas9 system into human cells (summarized in Table 1). Each method has advantages and disadvantages, and depending on the intended application and target cell type, the right delivery method should be selected. These methods can be based on viral delivery [51,52,53,54] or lipid vector delivery of the CRISPR/Cas9 system [55], but genome editing can also be facilitated through fusion of the Cas9 protein to cell-penetrating peptides [56], or by directly electroporating the CRISPR/Cas9 system into cells of interest [27,55,57,58]. Interestingly, recently, the mechanism HIV-1 employs to target CD4^+^ T cells has been repurposed as a tool to direct viral particles containing the Cas9 system specifically to cells expressing CD4, i.e., T cells [59], opening up new potential avenues with this addition to the CRISPR/Cas9 toolbox [60], e.g., in vivo gene editing of CD4^+^ T cells.

In the context of T cells, CRISPR/Cas9 has been used as a gene editing tool to study intracellular signaling pathways [61], to enhance T cell effector function [62], e.g., by modulating cytokine production [63], or redirecting T cell antigen-specificity [59], and to alter the expression of (membrane-bound) receptors, such as nutrient receptors [64], immune checkpoint receptors (e.g., PD-1 [65]) and chemokine and cytokine receptors (e.g., CCR5 [52]). Especially the latter is of interest in the context of HIV infection and will be further explored in the remainder of this manuscript. Importantly, autologous stem cells have been gene edited with CRISPR to lack expression of CCR5 and were subsequently successfully transplanted in a patient with HIV and acute lymphocytic leukemia [66]. However, CCR5 disruption was low [66], indicating the need for optimization.

## 4. Targeting CCR5 via CRISPR/Cas9-Mediated Genome Editing

As a co-receptor, CCR5 facilitates cellular entry for CCR5-tropic HIV-1 viruses [9,70], making it an attractive target for designing therapeutic approaches for HIV. Unsurprisingly, CCR5 has been targeted through a myriad of CRISPR-based approaches in different animal models [71,72], but also in human cell lines and primary human cells (summarized in Table 2). Early reports indicated low genome editing frequencies [22] and high off-target activity [73] in plasmid reporter studies. However, since then, new and efficient CRISPR protocols and tools have been published, and both primary cells and (progenitor) stem cells have been successfully edited (Table 2**)**.

For instance, CCR5 gene-edited primary human CD4^+^ T cells were found to be resistant to HIV-1 infection in vitro [24,52,53,74], recapitulating results obtained with other gene editing tools, such as TALENs and zinc finger nucleases [75,76]. Additionally, the CCR5Δ32/Δ32 mutation has been introduced in cell lines and primary human CD4^+^ T cells via CRISPR [51,77], with no off-target effects detected [51]. However, while ZFN-mediated CCR5-knockout CD4^+^ T cells have been shown to persist in vivo [76], these numbers can decline rapidly. Therefore, a more lasting therapeutic solution would be a bone marrow transplantation with gene-edited stem cells.

Several different types of stem cells have also been CCR5-gene edited. For instance, CRISPR-mediated CCR5 knockout was shown to be feasible in adipose stem cells (ASCs) [78,79]. ASCs possess hematopoietic potential [80,81], and as such could be used as a cellular source for therapeutic applications. However, more importantly, human HSCs were also successfully edited with CRISPR to lack CCR5 expression [82]. These CCR5-knockout HSCs retained multi-lineage engraftment potential in engraftment experiments [82].

This was expanded upon by Xu et al., where also in an in vivo animal model resistance to HIV infection was observed after CCR5-knockout HSC engraft generated via CRISPR/Cas9 [57]. As discussed, autologous CRISPR-edited CCR5 knockout HSCs have also been successfully transplanted in a patient with HIV and acute lymphocytic leukemia [66]. CCR5-knockout HSCs engrafted, resulting in host chimerism, with donor cells displaying the CCR5 ablation persisting for more than 19 months. No gene-editing-related adverse events were reported. However, the percentage of CD4^+^ CCR5-knockout cells in circulation was low (between approximately 2.5 and 5%). Therefore, more optimization is required in order to attain a higher knockout percentage.

However, obtaining both ASCs and HSCs requires an invasive procedure, and can pose risks to the patient. Another source of versatile stem cells are human induced pluripotent stem cells (iPSC). iPSCs can be generated from a multitude of tissues or bodily fluids, including skin and blood [88,89], which can be harvested with relative ease. Furthermore, iPSCs can be reprogrammed into hematopoietic cells, including cells from the erythroid, megakaryocytic and myeloid lineages [90]. Already in 2014, human induced pluripotent stem cells (iPSCs) had been genetically engineered with CRISPR to lack CCR5 expression [83]. These CCR5-mutant iPSCs were subsequently differentiated into monocytes/macrophages, which, compared to wild-type iPSC-derived macrophages, were resistant to in vitro HIV infection [83]. Similar results were replicated by other groups [84,85]. Of note, iPSCs are not used routinely as a source of stem cells, but are currently under investigation for many clinical trials [91], including a trial with iPSC-derived neural stem cells [92] and iPSCs as a source for blood components and blood cells (Trial NL8923, JPRN-jRCTa050190117 and JPRN-UMIN000015345). Indeed, their longevity and ex vivo manipulability make them an ideal source of stem cells for transplantation purposes as a curative strategy for HIV and should be further explored.

However, effects of genetic knockouts should be carefully considered to ensure patient safety. As a chemokine receptor, CCR5 has been shown to play a role in, amongst others, viral defense [93]. Several reports have claimed that CCR5Δ32/Δ32 increases the risk for symptomatic infection or fatal outcome in, e.g., Influenza and West Nile Virus, but also reports have been published that contradict this [93]. In contrast, the Δ32/Δ32 has also been implicated to be associated with protection against hepatitis B infection, while another report has indicated that it is associated with chronic disease [93]. These contradicting reports indicate that thorough investigation is difficult, potentially due to the low natural occurrence of the CCR5 Δ32/Δ32 mutation, but makes it all the more evident that careful investigation and long-term follow-up are due once trials with CCR5 Δ32/Δ32 or CCR5 knockout (iPSC-derived) HSCs are underway.

## 5. Targeting CXCR4 via CRISPR/Cas9-Mediated Genome Editing

Other critical co-receptors have also been identified next to CCR5, such as CXCR4, which is instrumental for CXCR4-tropic HIV strains [9,35]. CXCR4 has also been a target for gene editing purposes as a curative solution for HIV (summarized in Table 3), albeit at a lower rate compared to CCR5. Nonetheless, CXCR4 knockout was shown to be feasible in cell lines [86,87] and primary CD4^+^ T cells [58,94,95]. Furthermore, CXCR4 knockout in primary human CD4^+^ T cells conferred in vitro resistance to HIV infection [25,40,74].

In contrast to CCR5, no studies are currently available describing the use of CXCR4 knockout cells in murine models or humans. Of note, CXCR4 plays an important role in the migration of hematopoetic stem and progenitor cells (HSPCs), and is also involved in bone marrow retainment of these cells [96]. Currently, CXCR4 antagonists are on the market. Blocking the CXCR4/CXCL12 axis with Food and Drug Administration-approved antagonists resulted in the mobilization of HSPCs into the bloodstream [97]. Interestingly, the HPSCs that had exited the bone marrow due to CXCR4 antagonism showed superior engraftment in transplantation assays [97]. Together, this data indicates that knocking out CXCR4 can have profound effects in the context of stem cell transplantation, which should be carefully considered when applying this as a curative strategy in HIV infection.

## 6. Simultaneous Deletion of CCR5 and CXCR4 via CRISPR/Cas9-Mediated Genome Editing

In order to combat both CXCR4- and CCR5-tropic strains, a dual knockout strategy would be ideal. However, as multiplex gene editing can be complicated, few manuscripts have explored this avenue (summarized in Table 4). Nonetheless, it was shown that dual knockout of CXCR4 and CCR5 is feasible in primary human CD4^+^ T cells [54,98]. Furthermore, knocking out both chemokine receptors did not impact survival and proliferation [54,98], and allowed T cells to retain their in vitro cytokine production [27]. Importantly, double knockout also conferred in vitro resistance to HIV infection with both CXCR4- and CCR5-tropic HIV strains [27,54,98]. A study by Li et al. expanded upon this work, showing that dual knockout in primary human CD4^+^ T cells also conferred resistance to HIV infection in a humanized murine model. However, authors also showed that there was poor bone marrow engraftment of CXCR4/CCR5 double knockout T cells compared to control T cells. As a possible solution, authors speculate that overexpression of other chemokine receptors could counteract the poor engraftment.

## 7. Investigating Other Targets That Could Contribute to a Functional HIV Cure

CRISPR has also been used as a research tool in cell lines to further understand HIV biology and host proteins involved in the anti-viral response, or contributing to viral success [86,87]. Recently, a CRISPR-screen was performed to identify host proteins that interact with HIV in primary human T cells [40]. Here, authors identified 62 proteins that are so-called dependency factors, proteins that HIV requires in order to infect and/or propagate in cells. For instance, knockout of previously known HIV-1 interactors Cyclin T1 (CCNT1), Peptidylprolyl isomerase A (CYPA) and Lens epithelium–derived growth factor (LEDGF) reduced susceptibility to HIV-1 infection of primary human CD4+ T cells to approximately 20 percent of wild-type T cells. Potentially, the results from this screen can be exploited to identify other targetable or druggable candidates that can be exploited to render cells resistant to HIV-1 infection, which warrants further investigation.

## 8. Targeting the HIV Viral Reservoir

Upon successful anti-retroviral treatment, HIV becomes dormant and forms a latent reservoir [10,99]. This latent reservoir consists of cells that contain the replication-competent virus, but currently do not produce new viral particles [10]. However, this latent reservoir could be reactivated, for instance upon a lapse in therapy. Therefore, this latent reservoir is still considered a danger in infected individuals. Viral latency can also reverse upon T cell activation, which can be enforced with stimulatory agents such as phorbol 12-myristate 13-acetate, bryostatin or ingenol [1,100]. This reversal could also potentially lead to antigen presentation, offering opportunities to target these cells with, e.g., adoptive therapies [4]. However, these molecules are not suitable for use in vivo. Of note, a recent unreviewed pre-print utilized engineered bacteriophage nanoparticles to target CD4^+^ T cells in vitro, resulting in T cell activation and latency reversal [101]. While promising, these results need to be expanded upon and tested in animal models to validate in vivo usage. Potentially, the bacteriophage-derived nanoparticle could be modified to facilitate simultaneous delivery of the CRISPR machinery.

CRISPR could also be used to directly target the HIV-1 genome and remove it from infected cells, for instance by targeting the HIV viral promoter long terminal repeat [55]. Importantly, in vitro experiments showed up to 100% viral excision [55]. While currently not employed as a therapeutic strategy yet, the approach is interesting. Indeed, in contrast to CCR5/CXCR4 gene editing, the patient’s immune system does not need to be replaced with new cells, but could rather be edited in vivo. Furthermore, this approach is not specific to HIV subtypes, and could provide a broader solution. However, CCR5/CXCR4 disruption would protect patients from reinfection, while long terminal repeat excision would not, as viral entry would not be blocked. These benefits and disadvantages should be carefully considered before implementation into the clinic.

## 9. Considerations for Monitoring Cellular and Viral Compartments after CRISPR/Cas9-Mediated Genome Editing

Clinical trials employing CRISPR/Cas9-edited cells have included monitoring of (stem) cell engraftment, survival and/or proliferative/differentiative capacity, and the effect on viral load. Typically, this has been through the use of flow cytometry to assess the cellular compartment, while the viral load is determined via classical methods, i.e., qPCR [66]. However, both assessments can be combined via flow cytometry by making use of flow cytometry in situ hybridization, or Flow-FISH. Flow-FISH can be used for the characterization of T cells, e.g., to investigate effector function [102], delineation of the amount of HIV infected T cells [103] or determining the HIV translation-competent viral reservoir [104]. As this technique is compatible with T cell phenotyping to assess membrane marker expression [104], HIV Flow-FISH could increase the depth of flow cytometric sample analysis after cellular therapy and/or (stem) cell transplantation in this setting [4]. For instance, in contrast to conventional techniques only monitoring the total viral load, HIV Flow-FISH could provide (novel) in-depth insights into the infected cell population(s) [103,104,105], while simultaneously allowing monitoring of cellular persistence after genome editing. However, compared to conventional diagnostic tools, HIV Flow-FISH is a more specialized assay, which requires additional laboratory equipment and trained personnel. These downsides should be carefully considered before implementing this type of assay [4].

## 10. Conclusions and Outlook

In this manuscript, the current literature describing the use of CRISPR as a curative strategy for HIV by targeting the membrane-bound co-entry receptors CCR5 and CXCR4 is summarized. Both CXCR4 and CCR5 are promising targets in this context. Especially the targeting of CCR5 has been thoroughly explored, even resulting in a first-in-human use of CRISPR-mediated CCR5-gene edited HSCs [66]. While the results indicated that further optimization is required to induce sufficient CRISPR-mediated CCR5 knockout cells [66], CCR5 depletion via HSC transfer from donors with a non-functional receptor (CCR5Δ32/32) was shown to be curative [11,12,13]. Due to the low amount of naturally occurring CCR5Δ32/32 (approximately 1%), the replication of this mutation through CRISPR-mediated genome editing in human T cells is promising [51]. Future research should focus on replicating this mutation in stem cells, and should aim to determine whether this approach impacts bone marrow engraftment or lineage potential, or raises other safety concerns.

Another approach that is of interest is the simultaneous knockout of CXCR4 and CCR5, rendering cells refractive to infection by both CXCR4- and CCR5-tropic strains. As discussed, dual knockout is feasible in primary human T cells but results in poor engraftment in vivo in murine models [74]. According to the authors, this could potentially be remedied by repurposing other chemokine receptors to enhance bone marrow engraftment and/or survival. Another possibility would be the use of gene-edited stem cells, such as HSC, or iPSC-derived HSCs. Again, efforts should be undertaken to understand whether the deletion of these chemokine receptors affects stem cell homing, engraftment or lineage potential. Making use of humanized mice models can be a first step to determine potential in vivo effectiveness and (side) effects.

## Figures and Tables

**Table 1:** Different CRISPR-methods and their (dis)advantages.

Method	Advantage(s)	Disadvantage(s)	Used in HIV Research?	Reference(s)
Cell-penetrating peptides	Little cell manipulation required In-house production	Batch-to-batch differences	Yes	[56]
Chemical transfection	Little cell manipulation required	Not every method is suitable for every cell type	Yes	[55]
Electroporation	Extensive protocols available Adaptable to cell type of interest	Can be cytotoxic [63] Costly	Yes	[27,55,57,58]
Lenti/retroviral vectors	Inclusion of (fluorescent) selection marker	Low knock-out efficacy Genomic integration	Yes	[51,52,53,54]
(Lipid) nanoparticles	Highly adaptable to specific (researcher) needs	Complex to engineer	Yes	[55]
Ligand fusion tags	Cell-type specific	Cells need to express receptor	No	[67,68]
Virus-like particles	Targetable to cell type of interest Potential for in vivo use	Dependent on viral mechanisms for specific cellular targeting	Yes	[55,69]

**Table 2:** CRISPR-mediated gene editing of CCR5.

Target	Remarks	Reference(s)
ASCs	CRISPR-mediated KO feasible	[78]
	Enhanced CCR5 KO when employing two crRNAs	[79]
HSCs	Knockout confers in vitro resistance to HIV infection in differentiated macrophages	[74]
	Multi-lineage differentiation in vitro	[53]
	Minimal off-target modifications detected Multi-lineage engraftment potential in animal model	[82]
	Multi-lineage engraftment potential in animal model In vivo resistance to HIV infection	[57]
	Multi-lineage engraftment potential after autologous HSC transplantation Persistence of low frequencies of CCR5 knockout CD4^+^ T cells	[66]
iPSCs	No off-target modifications detected iPSC-derived monocytes/macrophages resistant to HIV infection	[83,84,85]
Primary CD4^+^ T cells	Low transduction efficiency with lentiviral vectors	[52]
	Knockout confers in vitro resistance to HIV infection	[24,52,53,74]
	Introduction of Δ32/Δ32 mutation No off-target modifications detected	[51]
Macrophage or T cell cell-lines	CRISPR-mediated KO feasible	[78,86,87]
	Introduction of Δ32/Δ32 mutation High fidelity screening method	[77]

**Table 3:** CRISPR-mediated gene editing of CXCR4.

Target	Remarks	Reference(s)
Primary CD4^+^ T cells	Knockout is feasible	[27,58,94,95]
	Knockout confers in vitro resistance to HIV infection	[25,40,74]
Macrophage or T cell cell-lines	Knockout is feasible	[86,87]
	Knockout confers minimal in vitro resistance to HIV infection	[74]

**Table 4:** CRISPR-mediated simultaneous gene editing of CCR5 and CXCR4.

Target	Remarks	Reference(s)
Primary CD4^+^ T cells	Dual CXCR4 and CCR5 knockout feasible	[54,98]
	No impact on survival and proliferation upon double knockout	
	Knockout confers in vitro resistance to HIV infection	[27,54,98]
	T cells retain in vitro cytokine production potential	[27]
	Knockout confers in vivo resistance to HIV infection in murine model Poor engraftment in murine model	[74]

## Data Availability

Not applicable.
